# Impact of Body Mass Index on In-Hospital Complications in Patients Undergoing Percutaneous Coronary Intervention in a Japanese Real-World Multicenter Registry

**DOI:** 10.1371/journal.pone.0124399

**Published:** 2015-04-14

**Authors:** Yohei Numasawa, Shun Kohsaka, Hiroaki Miyata, Akio Kawamura, Shigetaka Noma, Masahiro Suzuki, Susumu Nakagawa, Yukihiko Momiyama, Kotaro Naito, Keiichi Fukuda

**Affiliations:** 1 Department of Cardiology, Ashikaga Red Cross Hospital, Tochigi, Japan; 2 Department of Cardiology, Keio University School of Medicine, Tokyo, Japan; 3 University of Tokyo, Healthcare Quality Assessment, Tokyo, Japan; 4 Department of Cardiology, Saiseikai Utsunomiya Hospital, Tochigi, Japan; 5 Department of Cardiology, National Hospital Organization, Saitama National Hospital, Saitama, Japan; 6 Department of Cardiology, Saiseikai Central Hospital, Tokyo, Japan; 7 Department of Cardiology, National Hospital Organization, Tokyo Medical Center, Tokyo, Japan; 8 Department of Cardiology, Keiyu Hospital, Kanagawa, Japan; Shenzhen institutes of advanced technology, CHINA

## Abstract

**Background:**

Obesity is associated with advanced cardiovascular disease. However, some studies have reported the “obesity paradox” after percutaneous coronary intervention (PCI). The relationship between body mass index (BMI) and clinical outcomes after PCI has not been thoroughly investigated, especially in Asian populations.

**Methods:**

We studied 10,142 patients who underwent PCI at 15 Japanese hospitals participating in the JCD-KICS registry from September 2008 to April 2013. Patients were divided into four groups according to BMI: underweight, BMI <18.5 (n=462); normal, BMI ≥18.5 and <25.0 (n=5,945); overweight, BMI ≥25.0 and <30.0 (n=3,100); and obese, BMI ≥30.0 (n=635).

**Results:**

Patients with a high BMI were significantly younger (p<0.001) and had a higher incidence of coronary risk factors such as hypertension (p<0.001), hyperlipidemia (p<0.001), diabetes mellitus (p<0.001), and current smoking (p<0.001), than those with a low BMI. Importantly, patients in the underweight group had the worst in-hospital outcomes, including overall complications (underweight, normal, overweight, and obese groups: 20.4%, 11.5%, 8.4%, and 10.2%, p<0.001), in-hospital mortality (5.8%, 2.1%, 1.2%, and 2.7%, p<0.001), cardiogenic shock (3.5%, 2.0%, 1.5%, and 1.6%, p=0.018), bleeding complications (10.0%, 4.5%, 2.6%, and 2.8%, p<0.001), and receiving blood transfusion (7.6%, 2.7%, 1.6%, and 1.7%, p<0.001). BMI was inversely associated with bleeding complications after adjustment by multivariate logistic regression analysis (odds ratio, 0.95; 95% confidence interval, 0.92–0.98; p=0.002). In subgroup multivariate analysis of patients without cardiogenic shock, BMI was inversely associated with overall complications (OR, 0.98; 95% CI, 0.95–0.99; p=0.033) and bleeding complications (OR, 0.95; 95% CI, 0.91–0.98; p=0.006). Furthermore, there was a trend that BMI was moderately associated with in-hospital mortality (OR, 0.94; 95% CI, 0.88–1.01; p=0.091).

**Conclusions:**

Lean patients, rather than obese patients are at greater risk for in-hospital complications during and after PCI, particularly for bleeding complications.

## Introduction

Obesity is an independent risk factor of advanced cardiovascular disease and mortality [1–3]. Some previous studies have reported that obesity is associated with adverse cardiovascular events after percutaneous coronary intervention (PCI) [4–6]. However, various studies performed in Western countries have reported that obese patients have better short- and long-term outcomes after PCI than non-obese patients [7–15]. This phenomenon is well known as an “obesity paradox”, not only among patients with coronary artery disease (CAD), but also in those with heart failure [16,17]. However, the precise mechanisms of this phenomenon are still unclear [18–20]. Additionally, there are few data regarding the obesity paradox especially in Asian populations, because few studies have been conducted in Asia.

Patients with CAD in Asian countries have different characteristics compared with those in Western countries (e.g., older age, lower body mass index (BMI), frequently smoke, and have less traditional risk factors, except for diabetes mellitus) [21,22]. In addition, relationships between cardiovascular risk factors and cardiovascular disease may differ in Asian populations and Western populations [23]. In particular, one of the biggest differences between populations is physique. The average BMI in patients with CAD is remarkably lower in Asian countries compared with Western countries [21,22]. Moreover, the impact of BMI on the incidence of cardiovascular disease may differ in Asian populations and Western populations. Lu et al. reported higher hazard ratios per 5 kg/m^2^ BMI increase for coronary heart disease and stroke in Asian cohorts than in Western cohorts [3]. Previous studies have suggested that lower cut-off points for BMI should be adopted in Asian than in Western countries [23]. Furthermore, Japanese patients with CAD tend to have more bleeding complications during and after PCI compared with Western populations [21,22], and undergo complex procedures because surgical revascularization is less preferred by patients.

Because the risk profiles and procedural preference of Japanese patients with CAD differ from those in Western populations, investigation of the obesity paradox in Japan is important. This study aimed to investigate the impact of BMI on in-hospital complications in patients undergoing PCI in a Japanese multicenter PCI registry.

## Material and Methods

### Study design

The Japan Cardiovascular Database (JCD) is a large, ongoing, prospective, multicenter cohort study that was designed to record clinical background and outcome data for PCI patients in Japan [24–28]. Data for approximately 200 variables are continuously being collected in this study. Participating hospitals are instructed to record data from consecutive hospital visits for PCI and to register these data into an internet-based database system.

Entered data were checked for completeness and internal consistency. Quality assurance of the data was achieved through automatic system validation and reporting of data completeness, education, and training for dedicated clinical research coordinators specifically trained for the present PCI registry. The senior study coordinator (I.U.) and exclusive on-site auditing by investigators (S.K. and H.M.) ensured proper registration of each patient.

PCI with any commercially available coronary device was included. The decision to perform PCI was made according to the investigators’ clinical assessment of the patients. This study did not mandate specific interventional or surgical techniques, such as vascular access, use of specific stents, or closure devices.

Major teaching hospitals within the metropolitan Tokyo area were selected for this study. Patients were enrolled based on the individual PCI event, and all consecutive PCI procedures during the study period were registered, including cases of failure. Patients aged <18 years were excluded from the study.

The majority of clinical variables in the JCD are defined according to the National Cardiovascular Data Registry. This registry is sponsored by the American College of Cardiology for conducting comparative research to determine factors that can lead to disparities in PCI management. The National Cardiovascular Data Registry is a large PCI registry system with over 1,000,000 entries for ischemic heart disease and over 500,000 entries for PCI that were collected from more than 500 institutions in the United States [29].

### Information disclosure

The study protocol was approved by the institutional review board committee at Keio University, School of Medicine in Japan. All of the participants provided written informed consent for the present study. Before the launch of the JCD registry, information on the objectives of the present study, its social significance, and an abstract were provided for clinical trial registration with the University Hospital Medical Information Network. This Network is recognized by the International Committee of Medical Journal Editors as an “acceptable registry,” according to a statement issued in September 2004 (UMIN R000005598).

### Study population

We analyzed data from 10,788 consecutive patients who underwent PCI at 15 Japanese hospitals participating in the JCD-KICS registry from September 2008 to April 2013. For the present analysis, 646 patients were excluded because of missing data on basic information, including sex, height, and/or body weight. We divided the remaining 10,142 patients into four groups according to BMI. BMI was defined as weight in kilograms divided by the square of the height in meters. The National Heart, Lung, and Blood Institute and the World Health Organization have introduced a weight classification for BMI. According to this classification, patients with a BMI of 18.5–24.9 kg/m^2^ are considered normal, those with a BMI of 25–30 kg/m^2^ are considered overweight, and those with a BMI >30 kg/m^2^ are considered obese [4]. Patients were divided into four groups according to BMI in the present study: underweight, BMI <18.5; normal, BMI ≥18.5 and <25.0; overweight, BMI ≥25.0 and <30.0; and obese, BMI ≥30.0.

Clinical, angiographic, and procedural complications were prospectively entered into the JCD-KICS registry database. The choice of access site was based on the preference of the interventional cardiologist. Although the sizes of the sheath and guiding catheter were not protocol mandated in this cohort, the commonly used size was 6–8 Fr in transfemoral intervention, and 6 Fr in transradial intervention (TRI). All of the patients underwent periprocedural anticoagulation via heparin based on institutional dosing instructions during PCI. A bolus dose of 5000–10000 IU was usually administered and additional doses were provided based on an activated clotting time of > 300 seconds during PCI. We did not have a mandated protocol for hemostasis after the PCI procedures. Details of post-procedural management were left to the primary operators’ discretion. The recommended antiplatelet therapy was long-term 81 mg aspirin daily and a thienopyridine (75 mg clopidogrel or 200 mg ticlopidine daily). The loading dose of clopidogrel was 300 mg, and dual antiplatelet therapy was continued for at least 12 months after drug-eluting stent implantation, and 1 month after bare-metal stent implantation.

The endpoints were defined as in-hospital mortality and other complications. Complications were defined as all complications as follows: severe coronary artery dissection or coronary perforation; myocardial infarction after PCI; cardiac shock or heart failure; cerebral bleeding or stroke; and bleeding complications. Severe coronary artery dissection was defined as an intimal tear of the coronary artery, leading to impaired blood flow (final thrombolysis in myocardial infarction flow grade <3) on an angiogram. Myocardial infarction was defined as the new occurrence of a biomarker-positive myocardial infarction after PCI. Bleeding complications in this registry were further defined as those requiring blood transfusion, prolonging hospital stay, or causing a decrease in hemoglobin of >3.0 g/dL. Furthermore, bleeding complications were divided into puncture-site bleeding, retroperitoneal bleeding, gastrointestinal bleeding, genitourinary bleeding, or other bleeding. Hematomas >10 cm for femoral access or >2 cm for radial access also qualified as access site bleeding.

### Data analysis

Continuous variables are expressed as mean ± standard deviation (SD). Categorical variables are expressed as a percentage. Continuous variables were compared using the Student’s t-test, and the differences between categorical variables were examined using the chi-squared test. Univariate logistic regression analysis was performed to specify the odds ratio (OR) for overall complications, in-hospital mortality, and bleeding complications within 72 hours. Multivariate logistic regression analysis was then performed to investigate independent predictors for overall complications, in-hospital mortality, and bleeding complications. Variables in these models were selected based on univariate p values <0.05 and overall clinical significance. Variables that were entered in these models included age, sex, BMI, hyperlipidemia, diabetes mellitus, current smoking, previous PCI, previous myocardial infarction, previous heart failure, cerebrovascular disease, peripheral artery disease, chronic obstructive pulmonary disease, hemodialysis, ST elevation myocardial infarction (STEMI), non-STEMI, unstable angina, stable angina, cardiogenic shock, use of intra-aortic balloon pumping (IABP), TRI, transfemoral intervention, three-vessel disease, left main trunk lesion, type C lesion, chronic total occlusion, and use of a rotablator. All statistical calculations and analyses were performed using JMP version 10.0 (SAS Institute, Cary, NC, USA). A p value of <0.05 was considered statistically significant.

## Results

### Baseline clinical characteristics

**Fig 1:**
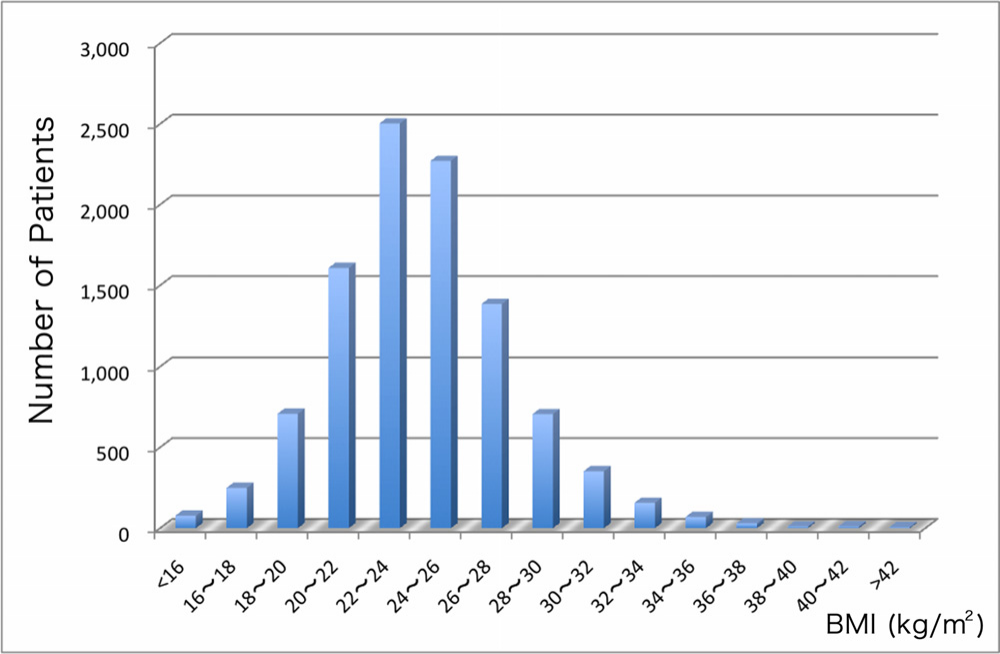
Distribution of BMI values. The distribution of BMI for 10,142 patients is shown.

The distribution of BMI for the 10,142 study patients is shown in Fig 1. The average BMI in the total cohort was 24.2. The baseline clinical characteristics of the study patients according to BMI are shown in Table 1. Of 10,142 patients, 4.5% (n = 462) were underweight, 58.6% (n = 5,945) were normal weight, 30.6% (n = 3,100) were overweight, and 6.3% (n = 635) were obese. Patients with a high BMI were significantly younger (p<0.001), had a higher incidence of coronary risk factors such as hypertension (p<0.001), hyperlipidemia (p<0.001), and diabetes mellitus (p<0.001), and had a higher rate of previous PCI (p<0.001) and current smoking habit (p<0.001) than those with a low BMI. On the other hand, patients with a low BMI were older (p<0.001), more often female (p<0.001), and more likely to have previous heart failure (p<0.001), peripheral artery disease (p<0.001), higher baseline serum creatinine level (p<0.001), and end-stage renal disease (p<0.001) than those with a high BMI. Furthermore, patients with a low BMI were more likely to present with acute coronary syndrome, especially with STEMI (p<0.001), unstable angina (p = 0.031) and cardiogenic shock (p<0.001) compared with those with a high BMI. The rate of administration of antiplatelet agents was not significantly different among the BMI groups.

**Table 1:** Baseline Clinical Characteristics.

	Underweight (BMI<18.5, n = 462)	Normal (18.5≦BMI<25.0, n = 5945)	Overweight (25.0≦BMI<30.0, n = 3100)	Obese (30.0≦BMI, n = 635)	P-value
Age, years	74.7±10.0	69.4±10.1	65.4±10.6	59.2±11.8	<0.001
Female gender	200(43.3%)	1233(20.7%)	512(16.5%)	135(21.3%)	<0.001
Height (cm)	157.9±9.8	161.7±8.7	163.1±8.6	164.1±9.5	<0.001
Weight (kg)	42.9±6.1	59.0±8.0	71.7±8.2	88.0±13.5	<0.001
Hypertension	317(68.6%)	4213(70.9%)	2481(80.0%)	539(84.9%)	<0.001
Hyperlipidemia	219(47.4%)	3801(63.9%)	2261(72.9%)	498(78.4%)	<0.001
Diabetes mellitus	156(33.8%)	2375(40.0%)	1438(46.4%)	372(58.6%)	<0.001
Insulin use	39(8.4%)	469(7.9%)	295(9.5%)	102(16.1%)	<0.001
Current smoking	121(26.2%)	1982(33.3%)	1201(38.7%)	284(44.7%)	<0.001
Family History	40(8.7%)	748(12.6%)	439(14.2%)	109(17.2%)	<0.001
Previous PCI	146(31.6%)	2316(39.0%)	1280(41.3%)	280(44.1%)	<0.001
Previous CABG	36(7.8%)	325(5.5%)	147(4.7%)	35(5.5%)	0.048
Previous HF	72(15.6%)	552(9.3%)	212(6.8%)	58(9.1%)	<0.001
Previous MI	121(26.2%)	1610(27.1%)	866(27.9%)	184(29.0%)	0.598
CVD	61(13.2%)	559(9.4%)	250(8.1%)	45(7.1%)	<0.001
PAD	77(16.7%)	518(8.7%)	193(6.2%)	33(5.2%)	<0.001
COPD	25(5.4%)	204(3.4%)	72(2.3%)	6(0.9%)	<0.001
STEMI	150(32.5%)	1423(23.9%)	628(20.3%)	132(20.8%)	<0.001
non-STEMI	42(9.1%)	482(8.1%)	220(7.1%)	52(8.2%)	0.255
Unstable angina	101(21.9%)	1098(18.5%)	551(17.8%)	96(15.1%)	0.031
Stable angina	76(16.5%)	1573(26.5%)	938(30.3%)	189(29.8%)	<0.001
Cardiogenic shock	39(8.4%)	239(4.0%)	83(2.7%)	26(4.1%)	<0.001
Serum creatinine (mg/dl)	1.8±2.3	1.3±1.9	1.2±1.7	1.3±1.9	<0.001
Hemodialysis	61(13.2%)	272(4.6%)	84(2.7%)	28(4.4%)	<0.001
Aspirin	442(95.7%)	5735(96.5%)	3022(97.5%)	615(96.9%)	0.113
Clopidogrel	325(70.4%)	4523(76.1%)	2378(76.7%)	492(77.5%)	0.084
Ticlopidine	15(3.3%)	183(3.1%)	110(3.6%)	29(4.6%)	0.311

Values are presented as n (%) or mean ± SD, as indicated.

BMI = body mass index; PCI = percutaneous coronary intervention; CABG = coronary artery bypass grafting; HF = heart failure; MI = myocardial infarction; CVD = cerebrovascular disease; PAD = peripheral artery disease; COPD = chronic obstructive pulmonary disease; STEMI = ST elevation myocardial infarction.

### Angiographic and Procedural Data

Angiographic and procedural data are shown in Table 2. TRI was performed more frequently in patients with a high BMI (p = 0.002). However, transfemoral intervention was performed more frequently in patients with a low BMI (p = 0.005). There was no difference in the frequency of use of a closure device in each group. The rotablator and IABP were used more frequently in patients with a low BMI than in those with a high BMI (p = 0.003).

**Table 2:** Angiographical and Procedural Data.

	Underweight (BMI<18.5, n = 462)	Normal (18.5≦BMI<25.0, n = 5945)	Overweight (25.0≦BMI<30.0, n = 3100)	Obese (30.0≦BMI, n = 635)	P-value
Two-vessel disease	142(30.7%)	1933(32.5%)	1007(32.5%)	233(36.7%)	0.135
Three-vessel disease	124(26.8%)	1432(24.1%)	751(24.2%)	169(26.6%)	0.315
Bifurcation lesion	127(27.5%)	1702(28.6%)	839(27.1%)	196(30.9%)	0.183
LMT lesion	20(4.3%)	258(4.3%)	87(2.8%)	22(3.5%)	0.003
CTO lesion	33(7.1%)	349(5.9%)	209(6.7%)	48(7.6%)	0.161
Type C lesion	163(35.3%)	1761(29.6%)	918(29.6%)	219(34.5%)	0.006
Transradial Intervention	116(25.1%)	1862(31.3%)	1026(33.1%)	218(34.3%)	0.002
Transfemoral Intervention	336(72.7%)	3939(66.3%)	2011(64.9%)	403(63.5%)	0.005
Drug-eluting stent	304(65.8%)	4183(70.4%)	2237(72.2%)	436(68.7%)	0.018
Sirolimus-eluting stent	22(4.8%)	349(5.9%)	173(5.6%)	31(4.9%)	0.584
Paclitaxel-eluting stent	24(5.2%)	208(3.5%)	117(3.8%)	24(3.8%)	0.303
Zotarolimus-eluting stent	47(10.2%)	487(8.2%)	272(8.8%)	47(7.4%)	0.307
Everolimus-eluting stent	176(38.1%)	2648(44.5%)	1393(44.9%)	275(43.3%)	0.044
Biolimus-eluting stent	37(8.0%)	445(7.5%)	231(7.4%)	49(7.7%)	0.973
Bare-metal stent	105(22.7%)	1363(22.9%)	670(21.6%)	158(24.9%)	0.265
Rotablator	32(6.9%)	251(4.2%)	108(3.5%)	21(3.3%)	0.003
Thrombus aspiration	104(22.5)	1195(20.1)	600(19.4)	139(21.9)	0.260
IVUS use	367(79.4%)	4803(80.8%)	2539(81.9%)	511(80.5%)	0.449
IABP use	46(10.0%)	465(7.8%)	191(6.2%)	40(6.3%)	0.003
Closure device	68(14.7%)	831(14.0%)	453(14.6%)	89(14.0%)	0.851

Values are presented as n (%) or mean ± SD, as indicated.

LMT = left main trunk; CTO = chronic total occlusion; IVUS = intravascular ultrasound; IABP = intra-aortic balloon pumping.

### Complications

**Fig 2:**
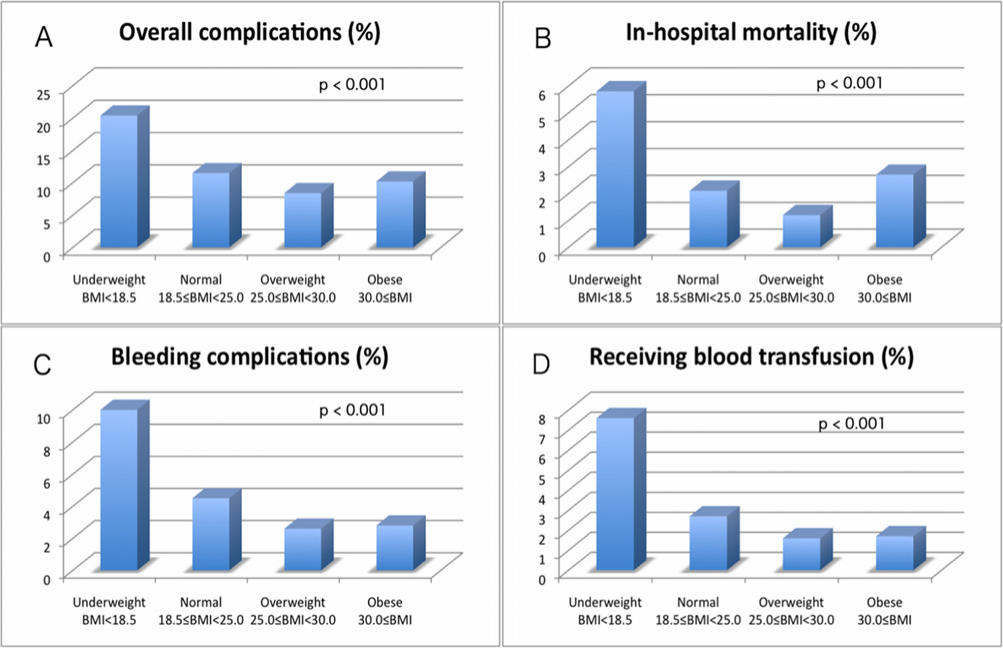
Relationship between BMI and in-hospital complications. In-hospital complication rates by BMI groups in 10,142 patients are shown. A: Overall complications; B: in-hospital mortality; C: bleeding complications within 72 hours; and D: rates of receiving blood transfusion.

In-hospital complications are shown in Fig 2 and Table 3. Importantly, patients in the underweight group had the worst in-hospital outcomes, including overall complications (p<0.001), in-hospital mortality (p<0.001), heart failure after PCI (p = 0.048), cardiogenic shock (p = 0.018), bleeding complications within 72 hours (p<0.001), and a higher incidence of receiving blood transfusion (p<0.001).

**Table 3:** Complications.

	Underweight (BMI<18.5, n = 462)	Normal (18.5≦BMI<25.0, n = 5945)	Overweight (25.0≦BMI<30.0, n = 3100)	Obese (30.0≦BMI, n = 635)	P-value
Overall complications	94(20.4%)	681(11.5%)	261(8.4%)	65(10.2%)	<0.001
In-hospital mortality	27(5.8%)	127(2.1%)	38(1.2%)	17(2.7%)	<0.001
Severe dissection	10(2.1%)	66(1.1%)	26(0.8%)	15(2.4%)	0.002
Coronary perforation	8(1.7%)	54(0.9%)	29(0.9%)	8(1.3%)	0.309
Myocardial infarction after PCI	10(2.2%)	135(2.3%)	61(2.0%)	13(2.1%)	0.819
Heart failure after PCI	10(2.2%)	121(2.0%)	40(1.3%)	8(1.3%)	0.048
Cardiogenic shock	16(3.5%)	117(2.0%)	45(1.5%)	10(1.6%)	0.018
Cardiac tamponade	3(0.7%)	22(0.4%)	6(0.2%)	1(0.2%)	0.244
Cerebral bleeding	3(0.7%)	2(0.1%)	1(0.1%)	0(0%)	<0.001
Cerebral infarction	5(1.1%)	23(0.4%)	11(0.4%)	1(0.2%)	0.086
Bleeding complications(<72h)	46(10.0%)	266(4.5%)	81(2.6%)	18(2.8%)	<0.001
Puncture site bleeding	13(2.8%)	66(1.1%)	25(0.8%)	5(0.8%)	0.001
Puncture site hematoma	7(1.5%)	60(1.0%)	25(0.8%)	2(0.3%)	0.154
Retroperitoneal bleeding	1(0.2%)	11(0.2%)	1(0.1%)	0(0%)	0.185
Gastrointestinal bleeding	2(0.4%)	24(0.4%)	8(0.3%)	3(0.5%)	0.687
Genitourinary bleeding	2(0.4%)	8(0.1%)	3(0.1%)	2(0.3%)	0.225
Other bleeding	12(2.6%)	64(1.1%)	20(0.7%)	4(0.6%)	0.001
New hemodialysis	5(1.1%)	53(0.9%)	26(0.8%)	5(0.8%)	0.951
Transfusion	35(7.6%)	163(2.7%)	49(1.6%)	11(1.7%)	<0.001

Values are presented as n (%).

PCI = percutaneous coronary intervention.

The results of multivariate logistic regression analysis on overall complications, in-hospital mortality, and bleeding complications within 72 hours in the total cohort are shown in Tables 4, 5 and 6. When BMI was entered as a continuous variable, it was not an independent predictor of overall complications (OR, 0.99; 95% CI, 0.97–1.01; p = 0.247) and in-hospital mortality (OR, 0.98; 95% CI, 0.94–1.03; p = 0.411), but was inversely associated with bleeding complications after adjustment for confounding variables (OR, 0.95; 95% CI, 0.92–0.98; p = 0.002). Because cardiogenic shock is known to be such a strong predictor of mortality and complications after PCI, we performed subgroup multivariate analysis in patients without cardiogenic shock (n = 9755, Tables 7, 8 and 9). In subgroup multivariate analysis, BMI was inversely associated with overall complications (OR, 0.98; 95% CI, 0.95–0.99; p = 0.033) and bleeding complications (OR, 0.95; 95% CI, 0.91–0.98; p = 0.006) after adjustment for confounding variables. Furthermore, there was a trend that BMI was moderately associated with in-hospital mortality (OR, 0.94; 95% CI, 0.88–1.01; p = 0.091).

**Table 4:** Multivariate Logistic Regression Analysis on Overall Complications in the Total Cohort.

	Univariate			Multivariate		
	OR	95% CI	p Value	OR	95% CI	p Value
Age	1.03	1.02–1.03	<0.001	1.02	1.01–1.03	<0.001
Female gender	1.62	1.41–1.87	<0.001	1.45	1.23–1.71	<0.001
BMI	0.94	0.92–0.95	<0.001	0.99	0.97–1.01	0.247
Previous HF	1.84	1.52–2.21	<0.001	1.52	1.22–1.89	<0.001
PAD	1.38	1.11–1.69	0.004	1.30	1.02–1.64	0.036
COPD	1.67	1.21–2.25	0.002	1.53	1.07–2.14	0.020
Hemodialysis	1.74	1.34–2.24	<0.001	1.48	1.09–1.97	0.011
STEMI	2.64	2.32–3.01	<0.001	2.08	1.66–2.61	<0.001
non-STEMI	1.42	1.14–1.74	0.002	1.45	1.09–1.92	0.010
Cardiogenic Shock	9.04	7.32–11.17	<0.001	2.39	1.84–3.09	<0.001
IABP use	9.73	8.27–11.45	<0.001	4.90	4.02–5.96	<0.001
Transradial Intervention	0.39	0.33–0.46	<0.001	0.61	0.39–0.98	0.040
Three-vessel disease	1.57	1.37–1.80	<0.001	1.19	1.02–1.39	0.024
Type C lesion	1.74	1.53–1.98	<0.001	1.41	1.21–1.64	<0.001
CTO	1.46	1.15–1.82	0.002	1.42	1.08–1.85	0.013
Rotablator	1.95	1.50–2.51	<0.001	1.68	1.25–2.24	<0.001

OR = odds ratio; CI = confidence interval; BMI = body mass index; HF = heart failure; PAD = peripheral artery disease; COPD = chronic obstructive pulmonary disease; STEMI = ST elevation myocardial infarction; IABP = intra-aortic balloon pumping; CTO = chronic total occlusion.

**Table 5:** Multivariate Logistic Regression Analysis on In-hospital Mortality in the Total Cohort.

	Univariate			Multivariate		
	OR	95% CI	p Value	OR	95% CI	p Value
Age	1.07	1.05–1.09	<0.001	1.07	1.05–1.09	<0.001
BMI	0.90	0.87–0.94	<0.001	0.98	0.94–1.03	0.411
Hyperlipidemia	0.35	0.27–0.46	<0.001	0.60	0.43–0.83	0.002
Previous HF	3.02	2.14–4.19	<0.001	2.42	1.53–3.76	<0.001
Hemodialysis	4.00	2.65–5.85	<0.001	7.31	4.14–12.66	<0.001
STEMI	5.65	4.27–7.53	<0.001	3.90	2.16–7.45	<0.001
non-STEMI	2.25	1.52–3.25	<0.001	2.96	1.51–6.01	0.002
Cardiogenic Shock	30.8	22.9–41.5	<0.001	5.74	3.89–8.50	<0.001
IABP use	24.5	18.4–32.9	<0.001	6.91	4.71–10.15	<0.001

OR = odds ratio; CI = confidence interval; BMI = body mass index; HF = heart failure; STEMI = ST elevation myocardial infarction; IABP = intra-aortic balloon pumping.

**Table 6:** Multivariate Logistic Regression Analysis on Bleeding Complications within 72 Hours in the Total Cohort.

	Univariate			Multivariate		
	OR	95% CI	p Value	OR	95% CI	p Value
Age	1.04	1.03–1.05	<0.001	1.01	1.00–1.03	0.011
Female gender	2.35	1.91–2.88	<0.001	2.13	1.68–2.69	<0.001
BMI	0.89	0.87–0.92	<0.001	0.95	0.92–0.98	0.002
Previous PCI	0.54	0.43–0.68	<0.001	0.70	0.52–0.94	0.016
Previous HF	2.14	1.62–2.78	<0.001	1.55	1.13–2.10	0.007
PAD	1.69	1.24–2.26	0.001	1.45	1.03–2.03	0.035
COPD	1.97	1.24–2.99	0.005	1.88	1.14–2.97	0.015
Hemodialysis	2.79	1.98–3.83	<0.001	2.14	1.45–3.11	<0.001
STEMI	2.27	1.85–2.77	<0.001	1.59	1.19–2.14	0.002
non-STEMI	2.09	1.56–2.75	<0.001	1.92	1.35–2.70	<0.001
Cardiogenic Shock	7.99	6.10–10.38	<0.001	2.45	1.74–3.42	<0.001
IABP use	7.64	6.11–9.51	<0.001	3.50	2.63–4.63	<0.001
Transradial Intervention	0.33	0.25–0.44	<0.001	0.55	0.29–1.12	0.551
Closure device	0.91	0.67–1.21	0.522			
CTO	1.64	1.16–2.27	0.006	1.78	1.20–2.61	0.005
Rotablator	2.63	1.84–3.67	<0.001	2.25	1.50–3.31	<0.001

OR = odds ratio; CI = confidence interval; BMI = body mass index; PCI = percutaneous coronary intervention; HF = heart failure; PAD = peripheral artery disease; COPD = chronic obstructive pulmonary disease; STEMI = ST elevation myocardial infarction; IABP = intra-aortic balloon pumping; CTO = chronic total occlusion.

**Table 7:** Multivariate Logistic Regression Analysis on Overall Complications in Patients without Cardiogenic Shock.

	Univariate			Multivariate		
	OR	95% CI	p Value	OR	95% CI	p Value
Age	1.03	1.02–1.04	<0.001	1.02	1.01–1.03	<0.001
Female gender	1.73	1.49–2.02	<0.001	1.49	1.26–1.76	<0.001
BMI	0.93	0.91–0.95	<0.001	0.98	0.95–0.99	0.033
Previous HF	1.80	1.46–2.20	<0.001	1.41	1.12–1.75	0.003
PAD	1.41	1.12–1.76	0.003	1.31	1.02–1.66	0.034
COPD	1.51	1.05–2.10	0.026	1.36	0.92–1.95	0.114
Hemodialysis	1.78	1.35–2.33	<0.001	1.43	1.05–1.92	0.025
STEMI	2.07	1.79–2.40	<0.001	2.02	1.69–2.40	<0.001
non-STEMI	1.32	1.03–1.65	0.025	1.38	1.06–1.77	0.017
IABP use	7.87	6.47–9.55	<0.001	5.57	4.52–6.86	<0.001
Transradial Intervention	0.43	0.36–0.51	<0.001	0.61	0.50–0.73	<0.001
Three-vessel disease	1.53	1.32–1.78	<0.001	1.15	0.98–1.35	0.087
Type C lesion	1.74	1.51–2.00	<0.001	1.44	1.22–1.69	<0.001
CTO	1.51	1.18–1.92	0.001	1.40	1.05–1.84	0.020
Rotablator	2.25	1.73–2.92	<0.001	1.66	1.23–2.21	0.001

OR = odds ratio; CI = confidence interval; BMI = body mass index; HF = heart failure; PAD = peripheral artery disease; COPD = chronic obstructive pulmonary disease; STEMI = ST elevation myocardial infarction; IABP = intra-aortic balloon pumping; CTO = chronic total occlusion.

**Table 8:** Multivariate Logistic Regression Analysis on In-hospital Mortality in Patients without Cardiogenic Shock.

	Univariate			Multivariate		
	OR	95% CI	p Value	OR	95% CI	p Value
Age	1.10	1.08–1.13	<0.001	1.09	1.07–1.12	<0.001
BMI	0.82	0.77–0.87	<0.001	0.94	0.88–1.01	0.091
Hyperlipidemia	0.40	0.27–0.58	<0.001	0.70	0.46–1.05	0.087
Previous HF	3.69	2.35–5.63	<0.001	2.47	1.46–4.06	0.001
Hemodialysis	5.40	3.24–8.61	<0.001	7.82	4.25–13.96	<0.001
STEMI	4.74	3.24–6.96	<0.001	6.12	3.78–10.11	<0.001
non-STEMI	1.93	1.07–3.25	0.030	2.74	1.38–5.21	0.005
IABP use	15.35	10.34–22.64	<0.001	9.01	5.81–13.91	<0.001

OR = odds ratio; CI = confidence interval; BMI = body mass index; HF = heart failure; STEMI = ST elevation myocardial infarction; IABP = intra-aortic balloon pumping.

**Table 9:** Multivariate Logistic Regression Analysis on Bleeding Complications within 72 Hours in Patients without Cardiogenic Shock.

	Univariate			Multivariate		
	OR	95% CI	p Value	OR	95% CI	p Value
Age	1.04	1.03–1.05	<0.001	1.02	1.01–1.04	<0.001
Female gender	2.29	1.77–2.96	<0.001	1.82	1.38–2.38	<0.001
BMI	0.89	0.86–0.93	<0.001	0.95	0.91–0.98	0.006
Previous PCI	0.60	0.45–0.78	<0.001	0.69	0.51–0.92	0.012
Previous HF	2.12	1.50–2.93	<0.001	1.55	1.07–2.20	0.021
PAD	1.27	0.82–1.88	0.273			
COPD	1.59	0.83–2.74	0.151			
Hemodialysis	2.97	1.97–4.34	<0.001	2.20	1.40–3.34	<0.001
STEMI	1.61	1.22–2.11	<0.001	1.65	1.19–2.29	0.003
non-STEMI	1.98	1.36–2.80	<0.001	1.97	1.31–2.89	0.001
IABP use	4.52	3.22–6.21	<0.001	3.07	2.15–4.32	<0.001
Transradial Intervention	0.37	0.26–0.51	<0.001	0.55	0.38–0.78	<0.001
Closure device	1.01	0.70–1.42	0.944			
CTO	2.06	1.38–2.99	<0.001	2.39	1.57–3.54	<0.001
Rotablator	3.42	2.29–4.95	<0.001	2.67	1.73–4.01	<0.001

OR = odds ratio; CI = confidence interval; BMI = body mass index; PCI = percutaneous coronary intervention; HF = heart failure; PAD = peripheral artery disease; COPD = chronic obstructive pulmonary disease; STEMI = ST elevation myocardial infarction; IABP = intra-aortic balloon pumping; CTO = chronic total occlusion.

Notably, variables that were independent predictors for overall complications, in-hospital mortality, and bleeding complications in the total cohort included age, previous heart failure, hemodialysis, STEMI, non-STEMI, cardiogenic shock, and use of IABP. TRI was an independent predictor of preventing overall complications (OR, 0.61; 95% CI, 0.39–0.98; p = 0.040). Use of a closure device was not a predictor of reducing bleeding complications by univariate analysis (OR, 0.91; 95% CI, 0.67–1.21; p = 0.522).

## Discussion

The major findings of this study were that lean patients, rather than obese patients, were at greater risk for in-hospital complications during and after PCI in one of the largest, contemporary, multicenter registries in Japan. Our dataset included more than 10,000 patients. This allowed us to analyze the various in-hospital outcomes in each BMI group.

One of the biggest differences between Japanese patients with CAD and those in Western countries is physique. The average BMI is remarkably lower in Japanese CAD patients compared with those in Western countries. Wang et al. reported a comparative study of Asian versus non-Asian White Americans with non-STEMI. In their study, BMI was significantly lower in Asian patients than in non-Asian White Americans (24.9 vs 27.8 kg/m^2^, p<0.001) [21]. Consistent with their study, the average BMI was 24.2 in our cohort. In addition, previous studies have reported that more than 70% of patients were overweight or obese in Western PCI registries [4,8,12]. However, only 36.8% of the patients (3,735/10,142) were overweight or obese in our study. Our data regarding BMI in Japanese CAD patients are consistent with previous studies in Japan [30,31]. Some previous studies have reported that patients with a BMI >40 kg/m^2^ are considered extremely obese [32–34]. However, because the present Japanese study group included only 18 patients (0.2%) with a BMI >40 kg/m^2^, they were included in the highest BMI subgroup [10]. Previous studies in Western countries have reported that lean patients and extremely obese patients are at greater risk for adverse outcomes after PCI [7,10,14,20,35,36]. In our study, complication rates showed a reverse J-shape relation with a peak in risk in the lowest BMI group (Fig 2), but not a bi-modal (U-shaped) relation with a peak in risk in the lowest and highest BMI groups, as observed in Western registries [20,35,36]. The small number of extremely obese patients in Japan is one of the major reasons for the unique obesity paradox that is observed in this country.

The precise mechanism of the obesity paradox remains unclear. However, there are some possible explanations for this phenomenon. Previous studies have shown that obese patients tend to have more aggressive and invasive therapy for CAD at a younger age [10,20,32,37]. Our data are consistent with those previous studies, and patients with a high BMI were younger than those with a low BMI in our study. Younger age at the time of PCI in patients with a high BMI may be one of the reasons for the obesity paradox [5,31]. However, Gruberg et al. reported that the relationship between BMI and mortality rate after PCI, and analysis by age groups showed that 1-year mortality rate was higher in patients with a normal BMI for all age groups, except for those younger than 50 years old [12]. Furthermore, obese patients tend to have a high rate of guideline-based optimal medical therapy, including statins, angiotensin-converting enzyme inhibitors, and beta-blockers [7,8,32]. Cessation of smoking, cardiac rehabilitation, and dietary counseling are more frequently enforced in overweight and obese patients than in lean patients [8,38]. Furthermore, patients with a low BMI tend to have more disease-induced cachexia induced by carcinoma, smoking, chronic obstructive pulmonary disease, chronic heart failure, and insulin-dependent diabetes mellitus. These comorbidities have been suggested as a possible explanation for the obesity paradox [10,12,16,17].

Potential overdosing of antiplatelets or anticoagulants, and differences in platelet biology have been reported as reasons for the high risk of bleeding with a low BMI [9,39]. Notably, in our study, almost all of the Japanese patients underwent PCI with a unified regimen of aspirin and clopidogrel during and after the procedure. This was because other agents, such as prasugrel and ticagrelor were not available at the time of this study in Japan. A unified regimen of antiplatelets for all CAD patients with various BMIs may affect the obesity paradox in bleeding complications after PCI [13]. Wang et al. reported that Asian patients with non-STEMI had a significantly higher risk of bleeding compared with non-Asian white patients [21]. They also reported that Asian patients were more likely to receive excess doses of antithrombotic agents compared with non-Asians. Appropriate doses of antiplatelets or anticoagulants may reduce bleeding complications. Furthermore, patients with a low BMI were older (p<0.001), more likely to have end-stage renal disease (p<0.001) and systemic atherosclerotic disease including cerebrovascular disease (p<0.001) and peripheral artery disease (p<0.001), than those with a high BMI in our cohort. These results are consistent with previous studies [9,13,14,40], and indicate that patients with a low BMI in our present study tended to have progressive atherosclerosis in the arterial system of the whole body. Arterial stiffness due to progressive atherosclerosis might be associated with a higher risk of bleeding in patients with a low BMI [9,14], although we performed multivariate analysis to adjust for possible confounding variables.

Because bleeding complications are associated with short- and long-term adverse outcomes after PCI [39,41], efforts for reducing bleeding complications are important. Bivalirudin, TRI, and use of a closure device are considered as bleeding avoidance strategies [42]. Bivalirudin is not available in Japan. Therefore, appropriate use of a closure device and TRI may be useful for reducing bleeding complications after PCI. In our study, there was no difference in the frequency of using a closure device in each BMI group, and use of a closure device was not a predictor of reducing bleeding complications by univariate analysis. TRI has been reported as a useful method for reducing bleeding complications compared with conventional transfemoral intervention [24,43]. In our study, TRI was an independent predictor of preventing overall complications in the total cohort. TRI was also associated with a small risk of bleeding in a subgroup analysis of patients without cardiogenic shock in multivariate logistic regression analysis. Approximately one-third of all of the PCIs in our dataset were performed with the transradial approach. Furthermore, TRI is performed more frequently in patients with a high BMI than in those with a low BMI [36]. Although TRI is more commonly performed in Japan than in Western countries [24], more frequent use of radial access in patients with a low BMI for reducing bleeding complications should be considered. Because patients with a low BMI have small vessels compared with patients with a high BMI, an unfavorable arterial sheath size has been reported as a possible explanation for increased access site bleeding complications in those with a low BMI [7,40,44]. Kang et al. reported that a high BMI was associated with a large diameter of the coronary arteries, and was associated with a large stent area after intravascular ultrasound-guided stent implantation [45]. They concluded that a high BMI is not associated with worse outcomes after drug-eluting stent implantation, despite more comorbidities, greater plaque burden, and more plaque rupture. A large vessel diameter in patients with a high BMI is one of the potential causes of the obesity paradox after PCI. Endovascular techniques and devices have evolved over the years, and smaller sheaths, guiding catheters, stents, and balloons have become available in recent years. However, physicians should be aware that lean patients are at greater risk for complications during and after PCI.

Obesity is an independent risk factor of advanced cardiovascular disease and mortality [1–3]. Although the obesity paradox may be a real phenomenon, physicians should be aware that patients with an increased body mass remain at high risk for development of CAD and poor outcomes over the long term [2,3,32]. Current guidelines recommend weight reduction to a BMI <25 as a second prevention for patients with CAD [46,47]. However, there is no clear evidence that a reduction in weight improves the prognosis of patients after PCI. Further long-term studies are needed in the future regarding this important issue.

### Study limitations

The first limitation of our study is that it was an observational clinical trial. The study population was heterogenous, including patients with different severities of coronary artery disease, ranging from acute coronary syndrome with cardiogenic shock to stable angina. Although we performed multivariate logistic regression analysis to adjust for possible confounding variables, some selection bias might not have been completely adjusted for in our statistical model, and the heterogeneity of the patients may have affected the incidence of complications in each of the BMI groups. Furthermore, we excluded 646 patients with missing data of basic information, including sex, height and/or body weight, which might have affected selection bias. Another limitation is that the duration of antiplatelet therapy and the size of the sheaths and guiding catheters were not recorded, and patients with cancer or other serious comorbidities were not excluded in our registry. These factors might have been associated with the rate of bleeding complications. Finally, the impact of BMI and in-hospital bleeding complications on long-term clinical outcomes in patients who undergo PCI should be investigated in our registry in the future.

## Conclusions

Lean patients, rather than obese patients are at greater risk for in-hospital complications during and after PCI, particularly for bleeding complications.
